# The NO-cGMP pathway participates in vascular adaptation to oxidative stress in Nrf2 KO mice

**DOI:** 10.1186/2050-6511-16-S1-A62

**Published:** 2015-09-02

**Authors:** Ralf Erkens, Christian M Kramer, Christina Panknin, Lisann Krause, Mathias Weidenbach, Thomas Krenz, Evanthia Mergia, Malte Kelm, Miriam M Cortese-Krott

**Affiliations:** 1 Cardiovascular Research Laboratory, Division of Cardiology, Pneumology and Angiology, Medical Faculty, Heinrich Heine University of Düsseldorf, Düsseldorf, Germany; 2 Institute for Pharmacology and Toxicology, Ruhr-University Bochum, Bochum, Germany

## Background

Endothelial dysfunction is considered both as cause and consequence of oxidative stress. Mice lacking the transcription factor nuclear factor (erythroid-derived 2)-like 2 (Nrf2 KO) can be considered as a model of chronic adaption of the organism to oxidative stress, as they miss a key master switch controlling the expression of antioxidant and protective enzymes involved in the regulation of the cellular redox state. In this study we aimed to investigate the coronary and vascular phenotype and systemic hemodynamics of these mice compared to wild type (WT) littermates.

## Results

We found that Nrf2 KO mice show an impaired left ventricular diastolic function as assessed by high resolution ultrasound. Accordingly, isolated perfused Nrf2 KO hearts showed an impaired response to β adrenergic stimulation by isoproterenol, while systolic left ventricular function was preserved. Surprisingly, blood pressure in Nrf2 KO mice was significantly decreased, and endothelial function of arterial conductance and coronary resistive vessels was preserved. This is consistent with an increased maximal dilation after vascular occlusion of the arteria iliaca externa, which indicates a fully preserved vascular and endothelial function in these mice. Mice lacking the endothelial nitric oxide synthase (eNOS KO) showed no dilatatory response to shear stress, confirming that flow-mediated-dilation response mainly depends on eNOS-dependent vasodilatory pathways. The circulating NO pool analysed by HPLC and chemiluminescence showed no differences between Nrf2 KO mice and WT littermates. However, western blot analysis revealed significant increased expression levels of eNOS and soluble guanylyl cyclase (sGC) in the aorta and the heart of Nrf2 KO mice compared to WT littermates. To verify the functionality of the eNOS/sGC pathway in the vessels, carbachol was applied to aortic rings to stimulate the eNOS-dependent synthesis of cGMP. Radioactive immunoassay showed elevated cGMP levels after stimulation indicating that eNOS/sGC pathway is up regulated in response to lack of Nrf2.

## Conclusion

Taken together, we here show that global knock-out of Nrf2 results in significantly impaired cardiac diastolic function, which is associated with a decreased myocardial relaxation, while systemic and coronary endothelial function are preserved. We conclude that the NO-cGMP pathway participates in vascular adaption to an imbalance in oxidative stress by increased eNOS and sGC expression, which results in a preserved systemic and coronary endothelial function.

